# Supplementary material for: Identification of photoperiod-induced specific miRNAs in the adrenal glands of Sunite sheep (Ovis aries)
Source: Front Vet Sci. 2022 Jul 22;9:888207. doi: 10.3389/fvets.2022.888207 (PMC9354845; doi:10.3389/fvets.2022.888207)
Supplement: Supplementary file 1 [file Data_Sheet_1.ZIP › Supplementary file/miRDeep2 file.zip!/miRDeep2 file/Know_Structure/SPLP42A2/oar-mir-323a.pdf]

miRBase precursor : oar-mir-323a
Total read count : 2243
oar-miR-323a-5p read count : 17
oar-miR-323a-3p read count : 2224
remaining reads : 2

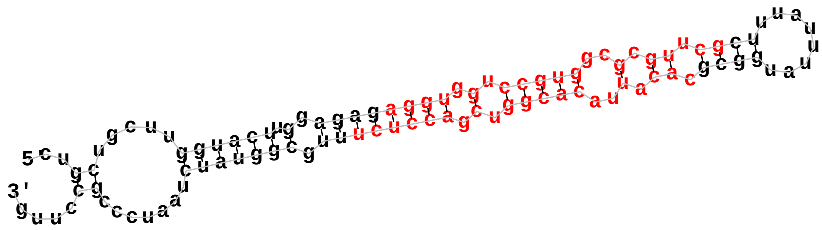

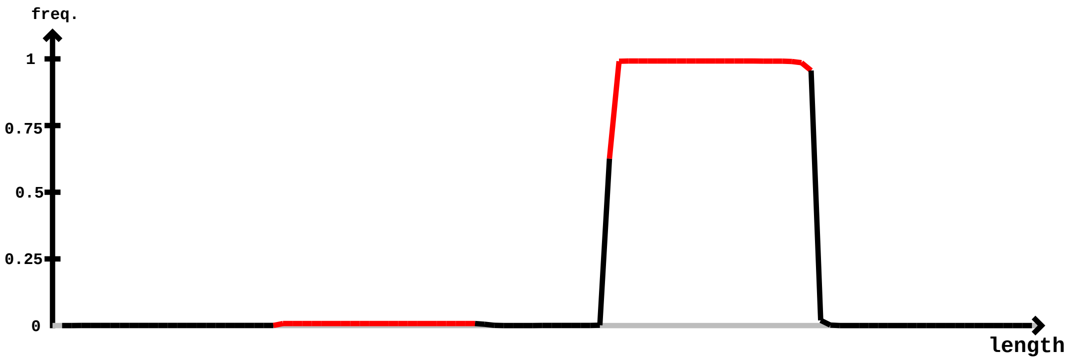

oar-miR-323a-5p

oar-miR-323a-3p

```
5'- cugcugcuugguacuuggagagaggugguccguggcgcguucgcuuuauuuauggcgcacauuacacggucgaccucuuugcgguaucuaaucccgccuug   -3'   exp
    .........(((((.(.((((((((.(.(((((..(.((.((((........)))).)).)..))))).).)))))))).))))))...............   reads   mm   sample
    .ugcugcuugguacuuggagag...............................................................................   1       0    seq
    ......................aggugguccguggcgcguucg..........................................................   6       0    seq
    ......................aggugguccguggcgcguucgc.........................................................   9       0    seq
    ......................aggugguccguggcgcguucgcu........................................................   2       0    seq
    .................................................uuauggcgcacauuacacggucg.............................   1       0    seq
    .......................................................cgcacauuacacggucgaccucu.......................   2       0    seq
    ........................................................gcacauuacacggucgacc..........................   3       0    seq
    ........................................................gcacauuacacggucgaccu.........................   5       0    seq
    ........................................................gcacauuacacggucgaccuc........................   38      0    seq
    ........................................................gcacauuacacggucgaccucu.......................   1335    0    seq
    ........................................................gcacauuacacggucgaccucuu......................   17      0    seq
    ........................................................gcacauuacacggucgaccucuuu.....................   3       0    seq
    .........................................................cacauuacacggucgaccu.........................   5       0    seq
    .........................................................cacauuacacggucgaccuc........................   28      0    seq
    .........................................................cacauuacacggucgaccucu.......................   762     0    seq
    .........................................................cacauuacacggucgaccucuu......................   23      0    seq
    .........................................................cacauuacacggucgaccucuuu.....................   1       0    seq
    ..........................................................acauuacacggucgaccucu.......................   2       0    seq
```
